# Effectiveness of two interventions based on improving patient-practitioner communication on diabetes self-management in patients with low educational level: study protocol of a clustered randomized trial in primary care

**DOI:** 10.1186/1472-6963-13-433

**Published:** 2013-10-23

**Authors:** Ignacio Ricci-Cabello, Antonio Olry de Labry–Lima, Julia Bolívar-Muñoz, Guadalupe Pastor-Moreno, Clara Bermudez-Tamayo, Isabel Ruiz-Pérez, Fermín Quesada-Jiménez, Enrique Moratalla-López, Susana Domínguez-Martín, Ana M de los Ríos-Álvarez, Pilar Cruz-Vela, Miguel A Prados-Quel, José A López-De Hierro

**Affiliations:** 1 Department of Primary Care Health Sciences, Health Services and Policy Research Group, NIHR School for Primary Care Research, University of Oxford, Oxford, England; 2 Escuela Andaluza de Salud Pública, Campus Universitario de Cartuja, Cuesta del Observatorio 4, Apdo. 2070, 18080, Granada, Spain; 3 CIBER de Epidemiología y Salud Pública (CIBERESP), Barcelona, Spain; 4 Hospital Universitario Virgen de las Nieves, Av Fuerzas Armadas, 2, 18014, Granada, Spain; 5 Centro de Salud Cartuja, Casería del Cerro, s/n, 18013, Granada, Spain

**Keywords:** Diabetes mellitus type 2, Primary care, Healthcare inequalities, Diabetes self-management, Quality of diabetes care

## Abstract

**Background:**

In the last decades the presence of social inequalities in diabetes care has been observed in multiple countries, including Spain. These inequalities have been at least partially attributed to differences in diabetes self-management behaviours. Communication problems during medical consultations occur more frequently to patients with a lower educational level. The purpose of this cluster randomized trial is to determine whether an intervention implemented in a General Surgery, based in improving patient-provider communication, results in a better diabetes self-management in patients with lower educational level. A secondary objective is to assess whether telephone reinforcement enhances the effect of such intervention. We report the design and implementation of this on-going study.

**Methods/Design:**

The study is being conducted in a General Practice located in a deprived neighbourhood of Granada, Spain. Diabetic patients 18 years old or older with a low educational level and inadequate glycaemic control (HbA1c > 7%) were recruited. General Practitioners (GPs) were randomised to three groups: intervention A, intervention B and control group. GPs allocated to intervention groups A and B received training in communication skills and are providing graphic feedback about glycosylated haemoglobin levels. Patients whose GPs were allocated to group B are additionally receiving telephone reinforcement whereas patients from the control group are receiving usual care. The described interventions are being conducted during 7 consecutive medical visits which are scheduled every three months. The main outcome measure will be HbA1c; blood pressure, lipidemia, body mass index and waist circumference will be considered as secondary outcome measures. Statistical analysis to evaluate the effectiveness of the interventions will include multilevel regression analysis with three hierarchical levels: medical visit level, patient level and GP level.

**Discussion:**

The results of this study will provide new knowledge about possible strategies to promote a better diabetes self-management in a particularly vulnerable group. If effective, this low cost intervention will have the potential to be easily incorporated into routine clinical practice, contributing to decrease health inequalities in diabetic patients.

**Trial registration:**

Clinical Trials U.S. National Institutes of Health, NCT01849731.

## Background

It has recently been estimated that the prevalence of type 2 diabetes mellitus (T2DM) in Spain is 13.8% (adjusted by age and gender), and half of those patients did not know that they were diabetic [1]. Diabetes leads to significant microvascular and macrovascular complications, which imply a major financial burden on health services. In fact, it is estimated that a patient with T2DM uses 2 to 6 times more direct resources than people of the same age and gender with other chronic diseases [2]. However, diabetes does not affect all population groups equally; differences have been described according to socioeconomic status, ethnicity and gender, [3-5]. Thus, one systematic review showed that socioeconomic position inequalities affect T2DM incidence, prevalence and mortality in Europe, especially among women [6]. Similarly, a WHO-sponsored multinational study on vascular disease in T2DM [7] found that the mortality rate in people diagnosed with diabetes belonging to the lowest social group was double the rate of those in the highest group.

The scientific evidence shows that patients with a lower socioeconomic position have worse health literacy, and this in turn has been associated with inadequate self-management of diabetes. This has been attributed to a poor knowledge of the disease itself and to barriers in the verbal communication process within general practitioners and patients, which in turn influences beliefs and attitudes towards T2DM [4,8,9]. The increasing awareness of the impact of social inequalities on diabetes has led to the design, implementation and evaluation of healthcare interventions aimed at improving diabetes care in socially vulnerable population. These interventions, frequently also known as quality improvement strategies, are characterised by their variable nature, being based on direct actions with patients, conducted in primary care settings and targeted at specific ethnic groups or people of low socioeconomic status [10-12]. In general, the aim of these interventions is to increase knowledge of the disease, promote the use of health services and encourage behavioural changes related to disease self-care [10-12]. Thus, improvement in doctor-patient communication has been associated with better health outcomes [13], because patients understand their treatment, receive feedback from healthcare staff, and doctors are more collaborative [14].

Two systematic reviews of interventions to improve quality of diabetes care [11,15] identified an intervention that received attention because of its favourable results and its simplicity and feasibility to be incorporated in routine clinical practice. In this intervention Chapin et al. [14] developed a 1-page form to provide feedback to both patients and providers and tested it in an inner-city internal medicine clinic. The form was designed to facilitate providers’ documentation of patients’ self-care activities and glycosylated hemoglobin values in a format tailored to the clinic’s low-literacy population. The intervention involved placing the form with brief written instructions into patient charts. The study results showed that 51% of patients who received this intervention improved their HbA1c levels, while in the control group an improvement was only found in 18%, representing a statistically significant difference between the two groups.

As a result of an additional systematic review of strategies to improve quality of diabetes care in vulnerable population conducted by members of our team we have identified the provision of rapid turnaround of HbA1c availability as one of the most effective strategies to improve diabetes care [16].

In our country, despite the recognition of social inequalities, there is little experience on interventions among diabetic populations with a low socioeconomic status [10-12]. That is why we decided to carry out an intervention in primary care specifically aimed at improving the quality of diabetes self-management in people with low educational levels, adapting the intervention carried out by Chapin et al. [14].

This paper describes the study methods and presents data on the recruitment. The discussion highlights the recruitment challenges we encountered and the trade-offs between realistic field conditions and experimental control in the context of this effectiveness trial.

## Methods/Design

### Study design and aims

This study used a cluster randomized design. The protocol of this study is registered in the Clinical Trials U.S. National Institutes of Health database (Trial registration number: NCT01849731). The aim is to evaluate an intervention with two levels of intensity, versus usual care, among T2DM patients recruited from the general practice setting in a socially disadvantaged community. A cluster randomised design was used being General Practitioners (GPs) the unit of randomisation (cluster) and patients the unit of analysis. Cluster randomisation was used to control for potential contamination that could arise from having intervention and usual care patients allocated to the same GP [17]. GPs were randomised to provide one of the two interventions or usual care. We hypothesised that the more intense intervention (Intervention “B”) will have the greatest effect on diabetes-related health outcomes, the less intense intervention (Intervention “A”) will have an intermediate effect, and the usual care will have no appreciable effect (control group “C”).

### Study population and setting

The study was conducted in the Primary Care Centre “Cartuja” has the highest percentage of disadvantaged people in the city of Granada (Andalusia, Spain) [18]. A high proportion of patients attending to the centre is characterised by a low educational and income level and a high unemployment rate. The primary care centre is attended by 9 GPs and all were invited to participate in the study. Because the study is being conducted at a single health centre, we decided to include all adult patients (>18 years) with a diagnosis of T2DM who had a low educational level (illiteracy or primary school education) and inadequate glycaemic control (HbA1c > 7%). Patients were excluded if they were unlikely to complete the study, if their physical or mental condition would make study follow-up impossible, or if they were participating in another study. Withdrawal criteria were voluntary abandonment on the part of the patient and non-compliance with study requirements (not attending one of the scheduled visits in the intervention, refusal to provide blood samples or to complete the questionnaires).

We included all patients diagnosed with T2DM who met the inclusion criteria at each of the visits and attended the clinic between February and November 2011.

### Sample size

The power was calculated assuming that at the end of the intervention 10% of the control group and 30% of intervention groups A and B would achieve improved diabetes control (with HbA1c values falling to below 7%). In order to provide a level of confidence of 95%, a power to detect significant differences between groups A and B was 59.4%, while the power to detect differences between groups B and control was 57.9%.

### Procedure

**Figure 1:**
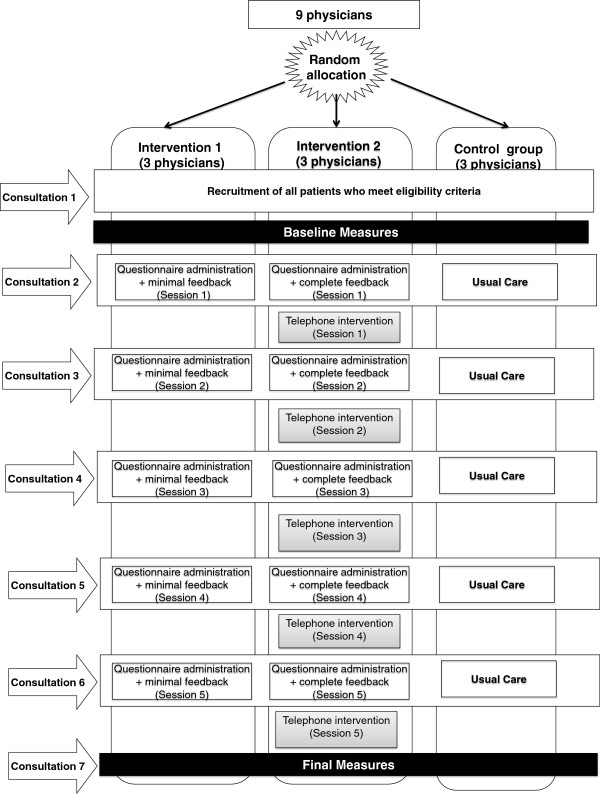
Study procedure.

After the Research Committee of the Andalusian School of Public Health had approved the trial, each patient was contacted in consecutive order. To facilitate recruitment, electronic medical records were searched for potentially eligible patients. During routine clinic visits they informed their patients of the study and invited them to participate. The study objectives were explained to the patient, who was then asked to sign the informed consent form. If a patient declined to participate, the next patient attending an appointment was asked to participate. If patients agreed to participate they completed a questionnaire to collect their socio-demographic characteristics, had a blood draw and were asked to return 14 days later to begin the intervention (see Figure 1).

### Interventions

The GPs interested in participating were assigned randomization to one of three intervention statuses: face-to face, face-to-face intervention plus telephone reinforcement and control group.

#### Intervention A

**Figure 2:**
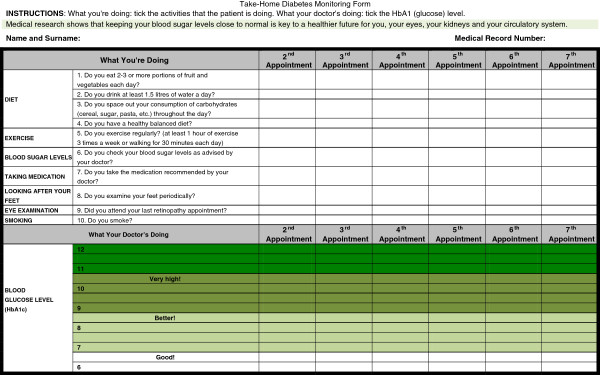
Diabetes care record sheet (DCRS).

(face-to-face intervention) is carried out by the GPs during the clinic visit and consists of seven visits, one every three months. Each session consists of completing a diabetes care record sheet (DCRS) together with the patient. The DCRS consists of two parts: Five questions on self-care activities in the last three months and a graph with previously measured HbA1c levels (see Figure 2). This information is completed at each session, resulting in a graph showing the evolution of glycaemic control related to self-care activities. The DCRS is explained patients, emphasising the relationship between self-care and glycemic control. At the end of the session, patients are given a copy of the DCRS and suggested to show it and discuss it with their relatives.

To standardise the intervention style, the nine general practitioners were trained in cognitive, emotional and communication aspects. In addition, every six months a joint meeting was held with the researchers and doctors to address complications, go over the intervention process and ensure standardisation of the interventions.

#### Intervention B

(face-to-face intervention plus telephone reinforcement) In this group patients receive the above described intervention A plus a telephone reinforcement It consists of five telephone calls lasting about 10 minutes each, to provide advice on carrying out physical exercise and eating a balanced diet and to encourage the use of health services related to diabetes control. Any problems or doubts that patients have stemming from any aspect of diabetes care are also discussed. Telephone reinforcement is carried out by a professional who has previously been trained in promoting T2DM self-management and in motivational interviewing techniques.

#### Control group

Patients pertaining to this group receive minimal intervention, which consists in establishing a rigorous follow-up every three months, including a measurement of HbA1c. Since patients who meet the selection criteria are being seen every three months for routine diabetes monitoring, it was decided to schedule all study phases (recruitment, baseline measurements, interventions and final measurements) to be carried out at the routine three-monthly appointments. This minimises the human and economic cost of the study as well as the burden on patients, and, in turn, will reduce withdraw rates. Therefore, after recruitment patients are given an appointment every three months for a period of 15 months (five appointments in total).

### Evaluation of effectiveness

Intervention effectiveness will be measured as the proportion of patients who achieve adequate glycaemic control (HbA1c of less than 7%) by the end of the study.

### Study variables

Our primary outcome is HbA1c, which is well established as a surrogate marker for the development of diabetes-specific complications. Secondary outcomes are blood pressure (systolic and diastolic values), lipidemia (triglycerides, high density lipoprotein and low density lipoprotein), body mass index and waist circumference. All of them are assessed at 3-monthly intervals. All clinical measures are collected at baseline, before each visit and 3 months after the final visit. Sociodemographic information were collected after obtaining informed consent and included age, gender, ethnicity, social support and number of children.

### Measurement tools

Social support is measured by Blake and McKay’s questionnaire [19]. “About how many friends or intimate or close relatives do you have? (People with whom you are comfortable and can talk about everything that happens)”. This question assesses the number of people available referring to a specific situation of tangible help. The category “low social support” means that the responder has 0 or 1 person as tangible aid, while “high social support” refers to having 2 or more such people. Finally, comorbidity is measured by asking the patient if he/she has any other disease apart from T2DM.

### Effectiveness assessment

Given the hierarchical structure of the information, the analyses will be performed using a multilevel model with three hierarchical levels: measurement (level 1), individual (level 2) and medical (level 3). The same levels of hierarchy will be used in the bivariate analyses, but adding a single independent variable to the model. All analyses will be carried out on an intention-to-treat basis.

### Trial status: recruitment and baseline characteristics

Table 1 shows the results of the first (sociodemographic data) and second (clinical data) appointments. Whenever a patient who met the inclusion criteria (age 18 years, low education and inadequate glycemic control) came to visit, the GP asked if they would collaborate in the study. Of all, 16 of them decided not to participate.

**Table 1:** Demographic and clinical characteristics of study subjects, by study group

		**All participants n =181 n (%)**	**Control group n = 92 n (%)**	**Intervention A n = 37 n (%)**	**Intervention B n = 52 n (%)**	**p**
Sex	Female	100 (54.3%)	44 (49.4%)	23 (63.9%)	31 (64.6%)	
	Male	80 (43.5%)	45 (50.6%)	13 (36.1%)	17 (35.4%)	0.143
Ethnicity	Caucasian	143 (80.8%)	77 (86.5%)	24 (66.7%)	36 (75%)	
	Romany/Other	34 (19.2%)	12 (13.5%)	12 (33.3%)	12 (25%)	0.033
Social support	High social support	44 (25.1%)	23 (25.6%)	2 (5.4%)	19 (39.6%)	
	Low social support	131 (74.9%)	67 (74.4%)	35 (94.6%)	29 (60.4%)	0.002
		**All participants Mean (SD)**	**Control group Mean (SD)**	**Intervention A Mean (SD)**	**Intervention B Mean (SD)**	**p**
Children		3.23 (2.16)	2.96 (3.33)	3.22 (3.69)	3.75 (2.16)	0.171
Age		61.66 (12.47)	62.37 (11.46)	60 (12.28)	61.59 (14.35)	0.721
HbA1c		8.77 (1.46)	8.57 (1.37)	8.74 (1.57)	9.16 (1.50)	0.031
Systolic pressure		130.63 (16.56)	127.90 (15.19)	128.43 (15.65)	137.16 (18.03)	0.004
Diastolic pressure		76.47 (11.32)	75.61 (9.41)	75.22 (11.04)	78.94 (14.18)	0.028
Abdominal circumference		107.44 (11.39)	105.73 (11.63)		111.16 (10.09)	0.030
HDL		47.64 (15.83)	48.66 (15.14)	46.43 (18.32)	46.64 (15.27)	0.378
LDL		122.82 (36.52)	125.40 (36.37)	120.64 (43.65)	119.56 (30.75)	0.453
Triglycerides		208.91 (165.13)	186.94 (89.40)	250.27 (258.79)	218.11 (179.53)	0.853
Body mass index		32.60 (5.72)	31.64 (5.40)	34.47 (5.50)	33.25 (6.17)	0.023

The sample is composed by a greater proportion of female patients (54.3%). The mean age of the sample is 61.99 years. Patients have an average of 3.23 children, and there are no significant between-group differences in these variables. Furthermore, 80.8% of the sample is Caucasian. A smaller proportion of this ethnicity is found in intervention group A (*p* = 0.033). Social support is low in the majority of the sample (74.9%), and the group B has a higher percentage of high social support (*p* = 0.002).

During the recruitment phase, one of the GPs did not arrange 14-day follow-up appointments for patients as stipulated in the study design. As a result, the baseline patient data used was from just 8 of the 9 GPs at the health centre. Although we considered replacing this GP, who declined to take part, with another similar one, in the end we could not do this for practical reasons.

Observed mean HbA1c value was 8.77%. For the control group this value was 8.57%, for intervention A it was 8.74% and for intervention B it was 9.16% (*p* = 0.031). For the lipid profile, the mean values of LDL, HDL and triglycerides were 122.82 mg/dL, 47.64 mg/dL and 208.91 mg/dL, respectively. There were no differences between the three groups. The mean systolic pressure was 130.63 mmHg (CG: 127.90; IA: 128.43 and IB: 137.16). The mean diastolic pressure was 74.47 mmHg (CG: 75.61; IA: 75.33 and IB: 78.94), with a significantly higher mean value in intervention group B. Likewise, significant differences were observed in the mean values of Body Mass Index, with 31.64, 34.47 and 33.25 kg/m2 being the mean values for the control group, intervention A and intervention B, respectively (*p* = 0.023).

## Discussion

This study has examined an intervention designed to improve self-management of diabetes in a group of patients with a low level of education. It describes the process, the results of the recruitment phase and the sample group’s baseline characteristics.

The sociodemographic profile of the patients in this study is similar to that of previous studies: a high percentage of patients in the group were women, from minority ethnic groups, and with little social support. The higher number of women in the group is consistent with the findings of other studies [14,20] despite the fact that T2DM is more prevalent amongst men. This may be because HbA1c levels are worse amongst women because they do not manage the disease as well, as found in other studies [21-24], or because women attend primary care appointments more frequently [25-27]. Furthermore, although only one study criterion related to social inequalities was used (level of education), the majority of the group was made up of women, ethnic minorities and people with little social support. This shows that social inequalities are often closely interlinked, making life even harder for these patients.

With regard to the clinical profile of patients in the study group, it is important to note that their test results were very high, higher than the recommended levels [28]. This is consistent with the literature on inequalities and with other interventions in disadvantaged groups and patients with diabetes [29-32]. We should also point out, however, that most of the test results were much more moderate in the control group than in the intervention groups. Because the sampling was done precisely, these differences will be taken into account in future analyses.

In order to carry out this study and choose the intervention type used, the authors carried out a systematic literature review of healthcare interventions that aim to improve diabetes care in socially vulnerable groups, identifying a total of 101 interventions (pending publication). Over recent years, a large number of interventions have been carried out in order to improve self-management of diabetes, and interventions aimed at socially disadvantaged groups have been found to be effective [10-12]. However, most of the interventions were carried out in the United States, a country with a healthcare system that does not offer healthcare services to the entire population. This is the first intervention in Spain specifically aimed at a diabetic population with a low level of education, and one of the few studies conducted in European countries. This study will provide new information about how to encourage satisfactory self-management of diabetes in a particularly vulnerable group, improving quality of care, quality of life and life expectancy, and reducing inequalities in diabetes care.

The study’s viability and methodological rigour were guaranteed because the intervention was planned by a multidisciplinary group of public health professionals with a good understanding of the methodological aspects of a study of this type. Furthermore, the study was carried out by primary care professionals with a good knowledge of the characteristics and habits of the reference population.

Another of the intervention’s strong points is its low cost. This is especially important today given the financial crisis and austerity measures affecting social welfare [33,34]. The intervention could also be easily transferred to other areas of the primary care system, which is the perfect setting given the high level of effectiveness of the intervention found in previous studies. In countries like Spain, which has its own national public health system, primary care is usually the first point of call for patients looking for healthcare services, and it has the potential to help reduce social inequalities in health [35,36]. T2DM is a very important disease because of its prevalence and cost, and although there are primary care guidelines in place, the results are not always satisfactory because effective management of the disease is hampered by difficulties [37]. Finally, the effectiveness of telephone reinforcement in interventions aiming to change patient behaviour has been widely proven. This technique is also very practical, because the patients do not need to attend the centre itself and telephones are widely available in primary care facilities [30,38,39].

There were a number of limitations to the study. Firstly, it was difficult to achieve the estimated sample size, and this could limit the statistical power to detect differences between the groups and the loss of patients during the follow-up period. Furthermore, this type of design may also be affected by bias in the standardisation of interventions. However, the professionals in charge of carrying out the two interventions were provided with training so that their interview styles would be as similar as possible, and joint meetings were organised in order to standardise the two intervention types as much as possible (in terms of intervention duration, empathy, emphasis and use of other communication skills). All of the doctors who took part in the study worked in the same health centre, so it was possible to standardise the population group.

The main intervention was carried out by healthcare staff during patients’ usual appointments, so the participation rate was high. The intervention was also designed to be as simple as possible in order to minimise the amount of work required from the doctors taking part. However, although the intervention was well-received by medical staff, the recruitment process was slower than anticipated, so the recruitment phase was extended to 5 months so that enough patients could be recruited for each group. Furthermore, there were recruitment problems during the initial stages with one of the GPs, and no other reason for this was found apart from the usual workload pressure. This shows that in order for healthcare staff to be able to devote extra time to carrying out studies of this type, it would be a good idea to combine the research work with their normal patient appointments. Additionally, consultation of the patients’ computerised clinical histories revealed an estimated prevalence of diabetes of approximately 900 patients, an average of 100 patients per GP, enough for the desired sample group size. Although the GPs were randomised, the patients who met the inclusion criteria were not evenly distributed across the different GPs, especially in terms of their level of education. Finally, a large number of patients left the study between the first and second appointments.

## Competing interests

The authors declare they have no competing interests.

## Authors’ contributions

IRC conceived the study, and led the design of the study with IRP, AOL, CBT and JBM. CBT was principal investigator. GPM took the lead role in data collection and analysis, and commented on drafts of the paper. Cartuja’s Primary Health Care Center physicians recruited patients and carried out the intervention. AOL led and conducted the quantitative data analysis. All authors interpreted the data and findings. IRC, JBM, AOL and GPM wrote the sections of the paper and all authors commented on it and all further revisions. All authors read and approved the content of this manuscript until the final version was approved.

## Pre-publication history

The pre-publication history for this paper can be accessed here:

http://www.biomedcentral.com/1472-6963/13/433/prepub
